# Periodontal Regeneration of Vital Poor Prognosis Teeth with Attachment Loss Involving the Root Apex: Two Cases with up to 5 Years Follow-Up

**DOI:** 10.3390/dj12060170

**Published:** 2024-06-05

**Authors:** Ethan Ng, John Rong Hao Tay

**Affiliations:** Department of Restorative Dentistry, National Dental Centre Singapore, 5 Second Hospital Ave, Singapore 168938, Singapore; john.tay.r.h@singhealth.com.sg

**Keywords:** guided periodontal tissue regeneration, prognosis, tooth extraction, health care economics, case report

## Abstract

Teeth with attachment loss involving the root apex are severely compromised and have a poor periodontal prognosis. In cases where periodontal regeneration is possible, current guidelines suggest that endodontic treatment is performed first. However, root canal treatment increases the overall treatment time and costs, has risks of endodontic complications, and could predispose teeth to mechanical failure. In this case report, two patients diagnosed with periodontitis stage III/IV grade C, no history of smoking or diabetes, and attachment loss involving the root apex of a tooth, were treated with guided tissue regeneration. These two cases are unique because successful periodontal regeneration was carried out without endodontic treatment, and the vitality of these teeth was maintained longitudinally. This report presents the management that led to this clinical outcome, and important guidelines for case selection are identified. Within the limitations of this study, vital teeth with radiographic bone loss involving the apex may be treated successfully with periodontal regeneration and remain vital at least in the short- to medium-term.

## 1. Introduction

Periodontal regeneration is the ultimate goal of periodontal therapy and is defined as the “restoration of lost or diminished periodontal tissues including cementum, periodontal ligament, and alveolar bone” [1]. Guided tissue regeneration (GTR), on the other hand, refers to a surgical procedure seeking to obtain the objectives of periodontal regeneration through utilising barrier devices or membranes, to exclude epithelial cells and provide space maintenance [1]. The clinical importance of GTR lies in the management of deep pockets associated with deep intrabony defects, with previous authors having classified such teeth as being ‘questionable’ at best [2], or ‘hopeless’ [3,4]. Indeed, deep residual probing pocket depths at the end of active therapy are positively correlated with an increased risk of tooth loss and disease progression [5]. Therefore, periodontal regeneration aims to improve tooth prognosis by increasing periodontal support and decreasing the probing pocket depth.

In the staging and grading system for periodontitis, periodontal regeneration remains the only modality that may result in the regression of a stage [6]. A recent systematic review conducted as part of the S3 treatment guidelines for periodontitis found that regenerative surgery with either enamel matrix derivative (EMD) or GTR led to superior clinical attachment level (CAL) gain than open flap debridement alone, and that this should be considered the treatment of choice for residual pockets with intrabony defects ≥ 3 mm [7]. Furthermore, other systematic reviews have found that post-treatment improvements following periodontal regeneration translate to higher rates of tooth survival, and these teeth are maintainable long-term with appropriate periodontal maintenance [8,9].

When teeth with attachment loss involving the apex show signs of altered pulp vitality, they are termed endo-periodontal lesions [10]. The current treatment option for such teeth is combined endodontic and regenerative periodontal therapy, with endodontic treatment carried out at least three months prior to re-evaluation [11,12]. In addition, the clinical strategies for the successful regeneration of teeth involving the apex have been previously presented in a comprehensive review, including the recommendation of endodontic treatment even for vital teeth when the defect involves the apex [13]. However, endodontic treatment adds another layer of treatment complexity, and complications can have an impact on the overall treatment outcomes [14]. The removal of the tooth structure during access cavity preparation may also compromise the mechanical integrity of teeth, and this is compounded by a higher maximal bite force from decreased sensitivity to occlusal loads after pulp removal [15]. This has been observed in a long-term cohort study of maintained periodontal patients with fixed prosthodontic treatment, where endodontic treatment and vertical root fractures accounted for the highest association with tooth loss [16]. Therefore, if a successful outcome can be achieved without endodontic treatment, there are potential benefits to the patient in terms of decreased costs, treatment time, overall complexity of treatment, and increased tooth longevity. This case report describes the successful periodontal regeneration in two patients with attachment loss involving the root apex, without endodontic treatment, adhering to CARE guidelines [17].

## 2. Materials and Methods

Two patients were referred from primary care for the management of periodontitis. They received Steps 1 and 2 of periodontal treatment, i.e., behavioural changes and risk factor control, followed by professional mechanical plaque removal [18]. After two post-instrumentation reviews over five to six months, non-responding sites were re-evaluated for surgical intervention. #46 ^‡^ (^‡^ Fédération Dentaire Internationale World Dental Federation notation) (case 1) and #36 (case 2) received guided tissue regeneration, and both surgical procedures were performed by the same clinician (EN). Endodontic treatment was not performed before the surgery, as both teeth consistently tested positive to cold and electric pulp tests and were clinically and radiographically asymptomatic for periapical pathology. Patient consent was obtained twice at two different time points. First, verbal consent was obtained after thoroughly discussing the proposed treatment plan, its non-standard nature, potential risks, and alternative treatment options with the patients. The treatment considerations were again discussed prior to written consent and proceeding with surgery. It was ensured that the patients fully understood the implications and voluntarily agreed to proceed.

After the surgical procedure, the patients were then reviewed at regular intervals, with documentation of the clinical and radiographic outcomes. This included maintenance of tooth vitality, post-operative reduction in probing pocket depths, gain in clinical attachment level, bleeding on probing, gingival recession, and radiographic bone fill. Digital periapical radiographs were taken with the long cone parallel technique and Rinn holders and assessed using anatomical landmarks, as previously described by Cortellini et al. [19]. Radiographic examinations were carried out by an examiner (JRHT) who was blinded to the procedures performed at each site. Radiographic bone fill was calculated as the percentage of the distance of the intrabony component bone fill over the distance of the original defect to the approximal bone crest, using the radiographic projection of the cemento-enamel junction as a fixed reference point. The detailed timeline for treatment is found in Tables S1 and S2.

### 2.1. Case 1

**Figure 1:**
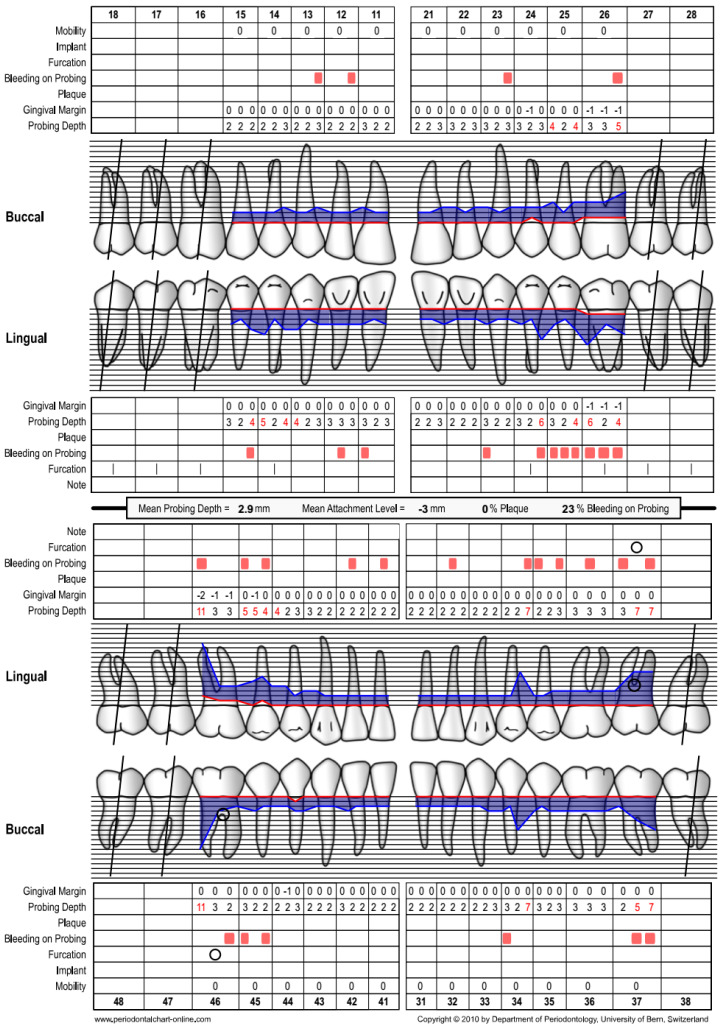
Case 1—Baseline periodontal chart.

**Figure 2:**
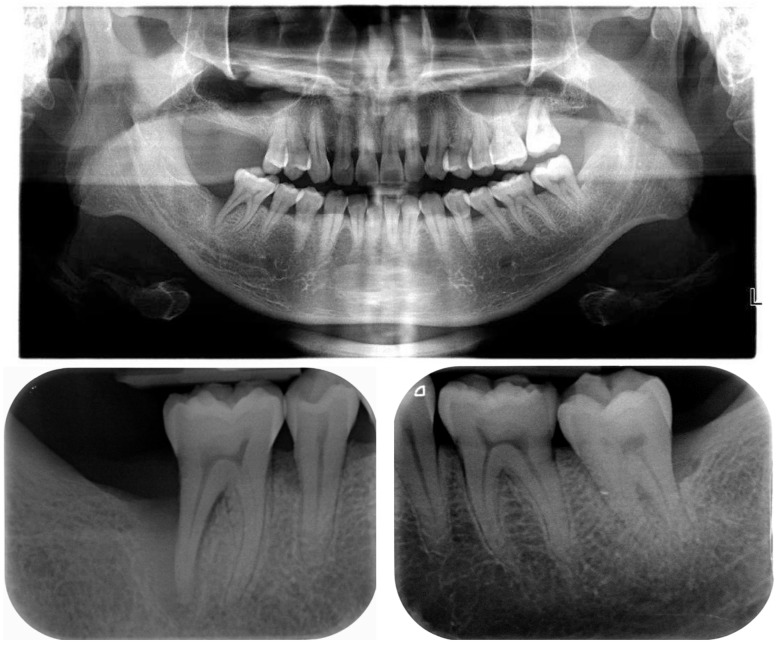
Case 1—Baseline radiographs. OPG taken in August 2018 at primary care, periapicals taken in October 2018.

A 41-year-old Chinese male patient was referred from primary care for periodontal treatment. On presentation, he had no immediate concerns as he was asymptomatic. The patient was a non-smoker, non-diabetic, and had hypertension which was under control with atenolol and losartan. He attended a private dental practice once a year, and had previously lost #17, #16, #47, and #27 due to pain and mobility. Clinical and radiographic examination revealed a diagnosis of periodontitis, stage III (localised) grade C (Figure 1 and Figure 2). #46 presented with bone loss involving the apex but was vital, asymptomatic, and did not display tooth mobility.

**Figure 3:**
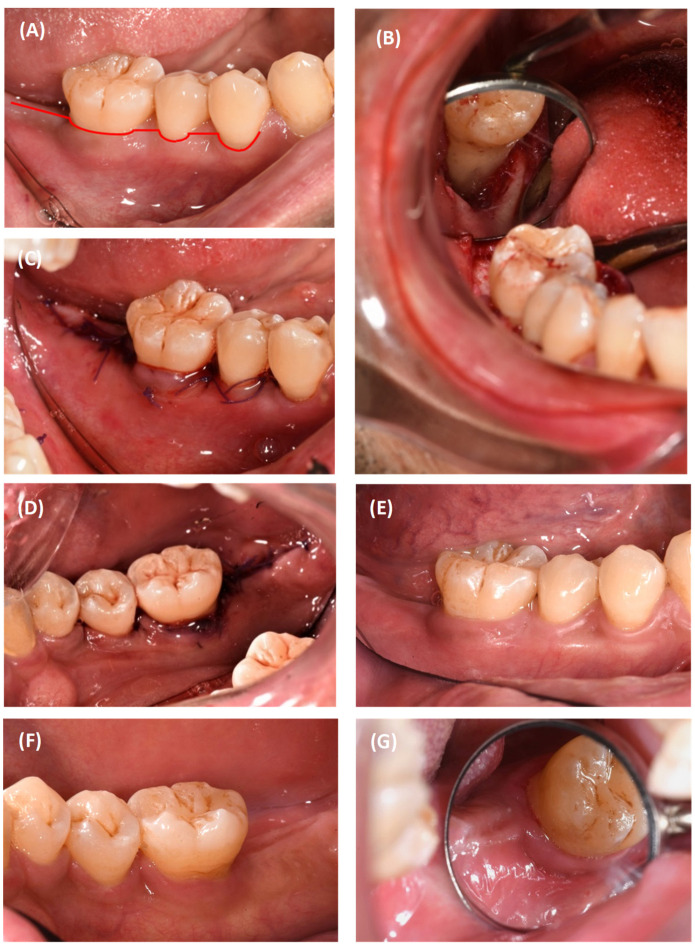
#46 surgical procedure: (**A**) incision design; (**B**) defect morphology revealing a 3-wall defect; (**C**) closure-buccal view; (**D**) closure-lingual view; (**E**) 5-year review-buccal view; (**F**) 5-year review-lingual view; (**G**) 5-year review-distal view.

Following administration of local anaesthetic (2% mepivacaine, 1:100,000 adrenaline), the flap design consisted of a modified papilla preservation double flap [20] (Figure 3A). Full-thickness flaps were raised, granulation tissue was removed, and the root surface was instrumented up to the root apex. The defect was contained, measuring 8 mm deep and 6 mm wide (Figure 3B). This was grafted with a deproteinised bovine bone mineral with 10% collagen (Bio-Oss^®^ collagen) and a resorbable collagen membrane (Bio-Gide^®^). Primary closure was achieved using a resorbable monofilament suture (Figure 3C,D). Post-surgically, the patient was prescribed analgesics (400 mg ibuprofen, three times daily, PRN) for five days, systemic antibiotics (500 mg amoxicillin, three times daily) for five days, and a chlorhexidine mouthwash for plaque control. The patient was seen at ten days for suture removal, once at three months for professional prophylaxis, and scheduled for six-monthly periodontal maintenance thereafter.

### 2.2. Case 2

**Figure 4:**
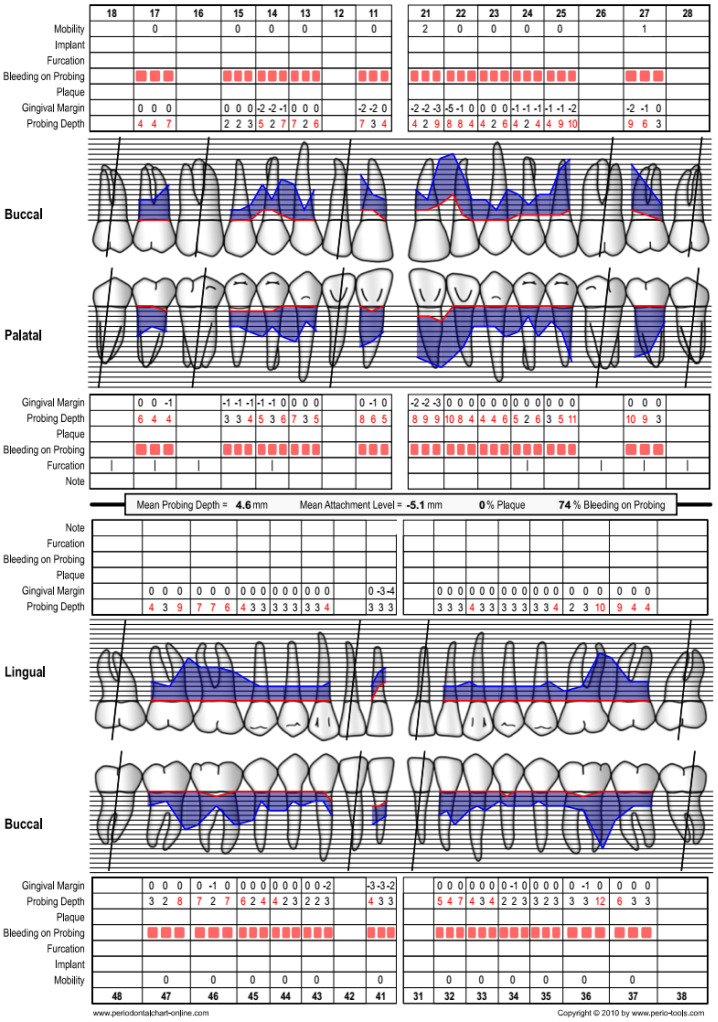
Case 2—Baseline periodontal chart.

**Figure 5:**
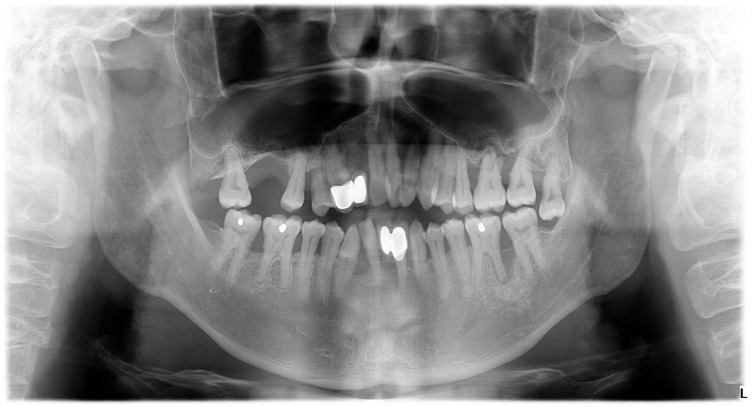
Case 2—Baseline radiographs. OPG taken in August 2020 at primary care.

A 40-year-old Chinese male patient had been referred for management of severe periodontitis. His presenting complaint was concern over his ‘gum problem’. The patient was a non-smoker, non-diabetic, and not on any medication. He saw a private dentist once every two years and had a positive familial history of gum disease. He had lost #16, #12, #26, #28, #31, and #42 due to periodontitis. Clinical and radiographic examination revealed a diagnosis of periodontitis, stage IV (generalised), grade C (Figure 4 and Figure 5). At baseline, #36 had bone loss involving the root apex but was vital, asymptomatic, and not mobile.

**Figure 6:**
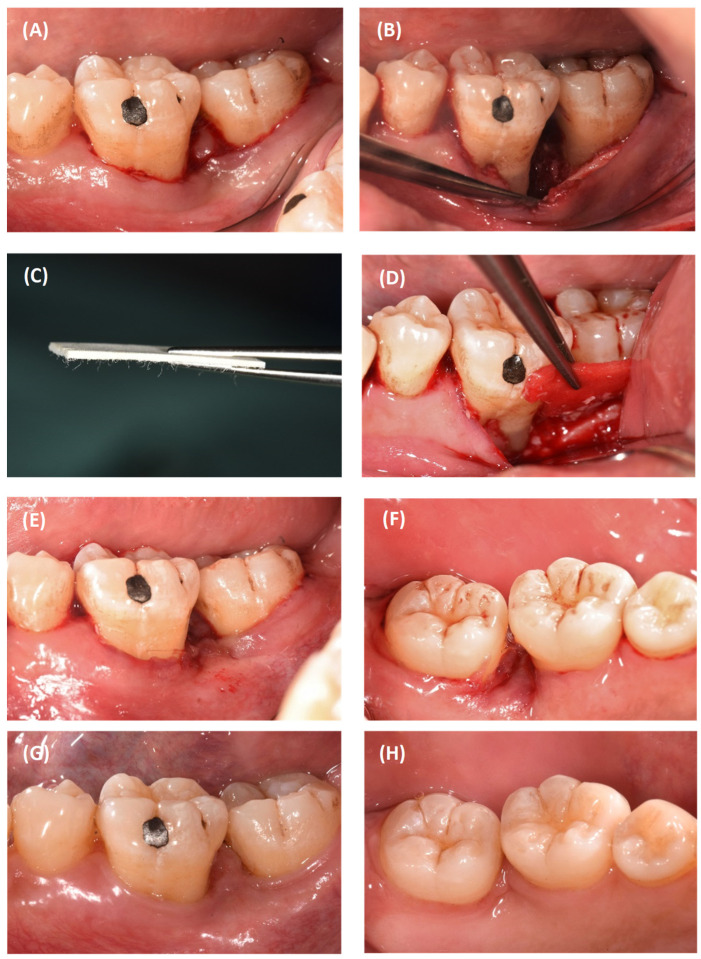
#36 surgical procedure: (**A**) incision design; (**B**) defect morphology revealing a 3-wall defect; (**C**) alloderm; (**D**) regenerative materials in place; (**E**) closure-buccal view; (**F**) closure-lingual view; (**G**) 3-year review-buccal view; (**H**) 3-year review-lingual view.

Following administration of local anaesthetic (2% mepivacaine, 1:100,000 adrenaline), the #36 defect was accessed with a modified papilla preservation double flap (Figure 6A). After removal of granulation tissue and instrumentation up to the root apex, a contained defect 6 mm wide and 5–8 mm deep was visualised (Figure 6B). As the patient was averse to animal products due to religious reasons, freeze dried bone allograft (SureOss^®^) and alloderm (SureDerm^®^) (Figure 6C) were used (Figure 6D). Primary closure was achieved using a resorbable monofilament suture (Figure 6E,F). Post-surgically, the patient was prescribed analgesics (400 mg ibuprofen, three times daily, PRN) for five days, systemic antibiotics (500 mg amoxicillin, three times daily) for five days, and a chlorhexidine mouthwash for plaque control. The patient was seen at ten days for suture removal, three-monthly for the first six months for professional prophylaxis, and scheduled for six-monthly periodontal maintenance.

## 3. Results

**Figure 7:**
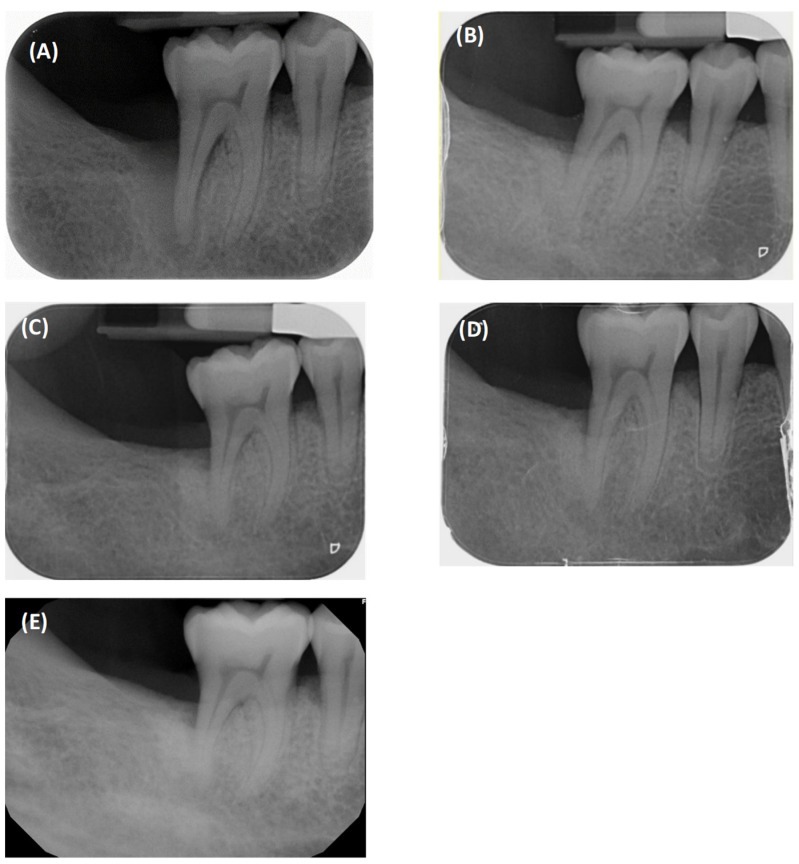
Radiographic follow-up of #46: (**A**) baseline; (**B**) 1 year; (**C**) 2 years; (**D**) 3.5 years; (**E**) 5 years.

The last recall for case 1 was at 5 years post-surgery (Figure 3E–G). The tooth remained vital and asymptomatic, and there was a sustained gain in the attachment level of 7 mm (Table 1). Radiographically, 71% bone fill was noted with a normal periapical region at 5 years (Figure 7).

**Figure 8:**
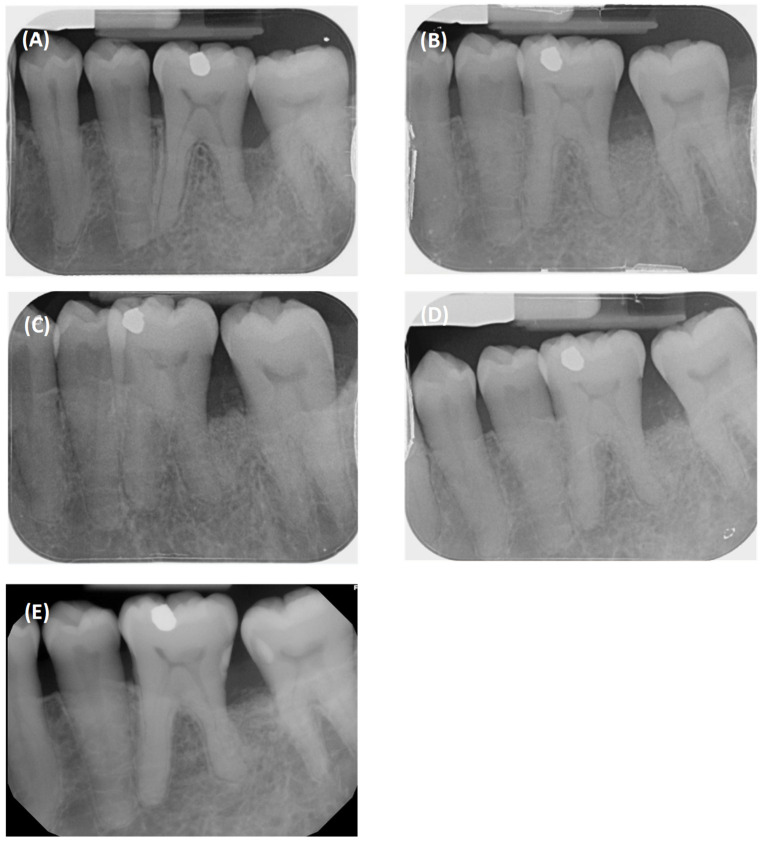
Radiographic follow-up of #36: (**A**) pre-op; (**B**) 6 months; (**C**) 1 year; (**D**) 1.5 years; (**E**) 3 years.

The final recall for case 2 was 3 years post-surgery (Figure 6G,H). The tooth remained vital and asymptomatic, and there was a sustained gain in attachment level of 8 mm (Table 2). Radiographic bone fill of 100% after the procedure was achieved and maintained up to the final recall at 3 years (Figure 8).

## 4. Discussion

These two cases describe the successful use of a regenerative procedure to significantly improve the prognosis and retention of strategically important teeth. Compared with other options such as root resection procedures, or extraction and implant placement, periodontal regeneration is considered more conservative, cost-effective, and minimally invasive [21]. In the present study, root canal treatment was not carried out despite the radiographic defect involving the root apex. This deviates from the treatment protocol of other studies, which recommends root canal treatment for non-vital teeth, teeth with inadequate root canal treatment, and vital teeth with defects beyond the apex [19,22,23]. The decision not to perform endodontic treatment was based on a few reasons. Firstly, the clinical and radiographic examinations showed no signs of pulpal or periapical pathology prior to periodontal surgery. Close monitoring over time also confirmed there was no development of endodontic complications or clinical symptoms such as pain or infection. Secondly, sensibility tests consistently indicated that the pulp was healthy and responsive. This has been described in the literature, where the pulp may remain vital even if the bacteria front is proximal to the apical foramina [24]. Thus, teeth may remain vital and overcome the bacterial insult after regenerative treatment. Thirdly, guided tissue regeneration in deep intrabony defects does not have a negative influence on the vitality of a tooth, nor does endodontic treatment negatively affect the healing response in guided tissue regenerative treatment [25]. Finally, the patients, being aware of the risks and benefits of performing endodontic treatment before periodontal regeneration, decided to proceed without endodontic treatment to potentially minimise costs. The patient accepted the risks that endodontic treatment might be required should the tooth subsequently devitalise, or tooth extraction should the regenerative procedure be unsuccessful.

The clinical and radiographic gains observed in this study are comparable to a previous multicentre randomised clinical trial on the treatment of deep and shallow intrabony defects [26]. The results obtained are also consistent with a previous clinical trial, where the successful periodontal regeneration of root canal-treated teeth was able to change the prognosis of ‘clearly hopeless teeth’ with deep intrabony defects extending to or beyond the root apex [19]. Longer term follow-up of the same study reported a survival rate of 92% at five years, and 88% at 10 years [22]. Another study reported a 92% five-year survival rate of endo-periodontal lesions successfully treated with periodontal regenerative therapy [12]. Like the present study, endodontic treatment was only performed if the tooth was unresponsive to sensibility testing, and this accounted for 15% of the teeth in that study. When there is endodontic involvement of the tooth, the emerging literature has shown that combined endodontic and regenerative periodontal therapy can also result in favourable tooth retention [11,27].

Periodontal regeneration can change the prognosis of ‘hopeless’ teeth and provides a less costly alternative to extraction and prosthetic replacement, resulting in cost-effective outcomes for the patient [28]. As observed in this study, clinically relevant and predictable results may be expected in the treatment of deep intrabony defects, while maintaining tooth vitality. Several factors contributed to the successful outcome in these cases. These include an accurate diagnosis, intervening early to prevent endodontic involvement, effective periodontal debridement, and preservation of the apical blood supply during surgery. Importantly, appropriate case selection is required, such as a vital and asymptomatic tooth beyond any doubt, no tooth mobility, and a contained defect (three walls) amenable to periodontal regeneration. The main strength of this study is that the results of these cases are unique, especially when no existing studies have compared the outcomes of periodontal regeneration with or without root canal therapy in teeth with defects involving the root apex. Another strength is the duration of follow-up, which also showed that the teeth did not subsequently de-vitalise after the procedure. The outcomes observed were also obtained despite the different biomaterials used. Notwithstanding, a clear limitation is that this is a report of only two cases. The longitudinal stability of clinical attachment gained also depends on a patient’s adherence to good oral hygiene and regular recalls [29]. While these initial cases provide promising results, a larger study is necessary to validate these findings and determine the reproducibility of this approach.

## 5. Conclusions

With appropriate case selection, vital teeth with radiographic bone loss involving the root apex may be treated successfully with periodontal regeneration and remain vital in the short- to medium-term. The observed clinical and radiographic gains can be maintained over up to 5 years.

## Figures and Tables

**Table 1:** Site-specific post-surgical follow-up of #46 clinical parameters compared to baseline.

	Baseline	3 Months	6 Months	1 Year	1.5 Years	2 Years	3 Years	3.5 Years	5 Years
Vitality	+	+	+	+	+	+	+	+	+
PPD (mm)	11	N/A	4	4	3	3	3	3	4
PPD reduction compared to baseline (mm)	N/A	N/A	−7	−7	−8	−8	−8	−8	−7
CAL (mm)	13	N/A	6	6	5	5	5	5	6
CAL gain compared to baseline	N/A	N/A	+7	+7	+8	+8	+8	+8	+7
BOP	+	N/A	−	+	−	−	+	−	+
GR (mm)	2	N/A	2	2	2	2	2	2	2
% bone fill	N/A	N/A	N/A	73	N/A	73	NA	73	71

PPD, probing pocket depth; CAL, clinical attachment level; BOP, bleeding on probing; GR, gingival recession; N/A, not applicable.

**Table 2:** Site-specific post-surgical follow-up of #36 clinical parameters compared to baseline.

	Baseline	3 Months	6 Months	1 Year	1.5 Years	3 Years
Vitality	+	+	+	+	+	+
PPD (mm)	14	N/A	5	5	5	5
PPD reduction compared to baseline (mm)	N/A	N/A	−9	−9	−9	−9
CAL (mm)	16	N/A	8	8	8	8
CAL gain compared to baseline	N/A	N/A	+8	+8	+8	+8
BOP	+	N/A	+	+	+	+
GR (mm)	2	N/A	3	3	3	3
% bone fill	N/A	N/A	100	100	100	100

PPD, probing pocket depth; CAL, clinical attachment level; BOP, bleeding on probing; GR, gingival recession; N/A, not applicable.

## Data Availability

The original contributions presented in the study are included in the article and Supplementary Materials, further inquiries can be directed to the corresponding author.

## Supplementary Materials

The following supporting information can be downloaded at: https://www.mdpi.com/article/10.3390/dj12060170/s1, Table S1. Timeline of treatment of case 1. Table S2. Timeline of treatment of case 2.
