# Supplementary material for: Periodontal Regeneration of Vital Poor Prognosis Teeth with Attachment Loss Involving the Root Apex: Two Cases with up to 5 Years Follow-Up
Source: Dent J (Basel). 2024 Jun 5;12(6):170. doi: 10.3390/dj12060170 (PMC11202695; doi:10.3390/dj12060170)
Supplement: Supplementary file 1 [file dentistry-12-00170-s001.zip › dentistry-3002963-supplementary.pdf]

Table S1. Timeline for treatment of case 1

Date	Appointment description
October 2018	Evaluation and diagnosis, baseline charting
November 2018	Initial periodontal therapy, oral hygiene instructions
January 2019	Periodontal review and re-instrumentation
April 2019	Second periodontal review and #46 GTR
July 2019	3/12 review and PMPR
October 2019	6/12 review and PMPR
March 2020	1-year review and PMPR
October 2020	1.5-year review and PMPR
February 2021	2-year review and PMPR
February 2022	3-year review and PMPR
October 2022	3.5-year review and PMPR
April 2024	5-year review and PMPR

GTR- Guided tissue regeneration, PMPR- Professional mechanical plaque removal

Table S2. Timeline of treatment of case 2

Date	Appointment description
November 2020	Evaluation and diagnosis, baseline charting, initial periodontal therapy, oral hygiene instructions
January 2021	Periodontal review and re-instrumentation
May 2021	Second periodontal review and #36 GTR
August 2021	3/12 review and PMPR
November 2021	6/12 review and PMPR
May 2022	1-year review and PMPR
November 2022	1.5-year review and PMPR
April 2024	3-year review and PMPR

GTR- Guided tissue regeneration, PMPR- Professional mechanical plaque removal
